# Cardiac Toxicity Associated with Immune Checkpoint Inhibitors: A Systematic Review

**DOI:** 10.3390/cancers13205218

**Published:** 2021-10-18

**Authors:** Walid Shalata, Amjad Abu-salman, Rachel Steckbeck, Binil Mathew Jacob, Ismaell Massalha, Alexander Yakobson

**Affiliations:** 1 The Legacy Heritage Center & Dr. Larry Norton Institute, Soroka Medical Center, Beer Sheva 84105, Israel; ismaell@post.bgu.ac.il (I.M.); alexy@clalit.org.il (A.Y.); 2 Cardiology Division, Soroka Medical Center, Beer Sheva 84105, Israel; amjadab@clalit.org.il; 3 Medical School for International Health, Ben Gurion University of the Negev, Beer Sheva 84105, Israel; steckbec@post.bgu.ac.il (R.S.); binil@post.bgu.ac.il (B.M.J.)

**Keywords:** cardiac toxicity, immune checkpoint inhibitors (ICIs), cardiotoxicity, cytotoxic T-lymphocyte-associated protein 4 (CTLA-4) inhibitors, programmed cell death protein 1 (PD-1), programmed death-ligand 1 (PD-L1), immune-related adverse events (IRAE)

## Abstract

**Simple Summary:**

This review article addresses the toxic effects on the heart associated with the use of certain cancer-treating drugs known as immune checkpoint inhibitors. These drugs target specific proteins in the cell cycle that are abundantly expressed in cancerous cells; however, they inadvertently damage non-cancerous tissue. In the heart, this occurs in the form of dysfunction or death of smooth muscle cells, leading to consequences such as infection, heart rhythm changes, and hormonally dependent and independent ischemia. This review examines the average and median onset of these drug toxicities as well as antidotes. One key observation is that these side effects are positively skewed, meaning they occur early in cancer treatment.

**Abstract:**

Immune checkpoint inhibitors are immune stimulatory drugs used to treat many types of cancer. These drugs are antibodies against inhibitory proteins, such as CTLA-4 and PD-1/PD-L1, that are expressed on immune cells. When bound, they allow for increased stimulation of T cells to fight tumor cells. However, immune checkpoint inhibitors have several immune-related adverse effects. Many cases have come to light recently of cardiotoxicity as a result of treatment with these drugs. Cardiotoxicity from immune checkpoint inhibitors is unique due to its rarity and high mortality rate. Patients with this toxicity may present with myocarditis, pericarditis, Takotsubo cardiomyopathy, conduction disorders, and others within just a few weeks of starting immune checkpoint inhibitors. We present here a review of the current research on immune checkpoint inhibitors, their associated cardiotoxicities, the timing of presentation of these conditions, lab tests and histology for each condition, and finally the treatment of patients with cardiotoxicity. We observe a positive skew in the onset of presentation, which is significant for the treating physician.

## 1. Introduction

In the last several years, immune checkpoint inhibitors (ICIs) have become the backbone of the treatment plan in many types of cancer. Tumor cells express neoantigens, mutated proteins that immune cells can recognize as foreign and destroy; however, many tumors also express factors that inhibit the immune system, thus allowing them to grow undetected in the body [1]. Immune checkpoint inhibitors work by blocking the inhibitory signals from tumor cells to T cells that recognize them, thus allowing the tumor cells to be destroyed by the patient’s own immune system. The first ICI was approved by the FDA in 2011 [2], and since then several different ICIs against a variety of targets have been approved. These include monoclonal antibodies against programmed death-1 (PD-1), such as nivolumab or pembrolizumab; ligands of PD-1 (PD-L1), such as atezolizumab; and cytotoxic T-lymphocyte-associated antigen-4 (CTLA-4), such as ipilimumab. These antibodies have complementary mechanisms of action to one another, and so they are often used in combination.

CTLA-4 is a marker expressed on activated T cells and regulatory T cells [3]. Competing with the stimulatory molecule B7, it binds to CD80 and CD86 on antigen presenting cells with high affinity, leading to inhibition of T cell proliferation and activity [3]. Blocking CTLA-4 with ipilimumab allows T cells to fight a tumor more effectively by removing inhibitory signals. The PD-1/PD-L1 system works very similarly; PD-1 on T cells binds to PD-L1, which is widely expressed on antigen-presenting cells as well as the tissues of the heart, muscle, lung, pancreas, and many more [4]. PD-L1 is considered essential to prevent autoimmunity in these tissues; lack of PD-1 leads to increased survival, proliferation, and killing capacity of T cells, which can cause autoimmunity [5]. PD-1/PD-L1 signaling also promotes the proliferation of regulatory T cells, further protecting against autoimmunity [4]. Importantly, some tumors can express PD-L1, thus inhibiting host immune responses against them [4]. Thus, blocking the PD-1/PD-L1 interaction through antibodies promotes immune reactions against tumor cells.

Though ICIs are effective in treating cancer; they have adverse effects distinct from cytotoxic chemotherapy because they directly affect the immune system. Immune-related adverse events (IRAEs) happen in 70–90% of patients treated with ICIs, with severe IRAEs happening in 10–15% of patients [6]; these reactions are fatal in up to 1.3% of patients [7]. Often, IRAEs occur within 1 year after treatment [8], but the risk of developing any IRAE increases with time [9]. These toxicities could be due to several causes: pre-existing self-reactive T cells that were inhibited becoming uninhibited, cross-reactivity between the tumor antigen and self-antigen, or T cells targeting a different but homologous antigen in the body as compared to the one on the tumor [6]. Signs and symptoms of ICI toxicity manifest as colitis, hepatitis, thyroiditis, hypophysitis, myo- or pericarditis, arthritis, uveitis, pneumonitis, or skin rash [8,10].

Cardiotoxicity due to ICIs is rare, with an incidence of up to 1% [11], but it is often severe and can be life threatening. Patients can present with cardiac fibrosis, cardiac arrest, autoimmune myocarditis, cardiomyopathy, heart failure, pericardial involvement, and vasculitis [10,11].

Here, we review immune checkpoint inhibitors, the cardiotoxicities they can cause, and the treatment of each cardiotoxicity. Though there have been multiple reviews on this subject already, there has been no research on the variations of time from beginning of treatment to the presentation of cardiotoxicity depending on the type of cardiotoxicity, the treatment, or the cancer being treated. In this review, we take special note of these differences and qualitatively evaluate them.

## 2. Materials and Methods

Multiple searches were performed on PubMed to obtain the studies used in this review, performed from 20 June 2021 to 31 July 2021. Search terms included “pericarditis with immune checkpoint inhibitors”, “cardiac toxicity immune checkpoint inhibitors”, and “myocarditis immune checkpoint inhibitors”, with results displayed from the last 10 years. The search terms were chosen to best find the broadest range of published papers on the topic of cardiac toxicity; in addition, myocarditis and pericarditis were searched for by name because they are the two most common cardiotoxicities that patients present with. From the search results, we reviewed all the papers displayed and found 134 relevant case studies and literature reviews.

All case reports were synthesized into a table. Of the case reports, all were published between the years 2015 and 2021, with the majority published between the years of 2018–2021. Case studies were segregated based on the specific antibody, the type of cardiotoxicity, and the type of cancer being treated. Trends were enumerated qualitatively through a table and a graph.

## 3. Cardiotoxicity

### 3.1. General

Cardiotoxicity is a very rare complication of immune checkpoint inhibitors, affecting up to 1% of patients; this percentage is higher in patients taking a combination of ICIs [11]. Others have argued that the total risk of cardiac events in patients with ICI therapy is much higher, from 3.1% [12] to 9.7% [13]. This discrepancy may be due to misclassification and difficult diagnosis of cardiac events caused by ICIs, especially during the current COVID-19 pandemic [14]. The most common presentation of cardiotoxicity due to ICIs is myocarditis (Table 1).

**Figure 1:**
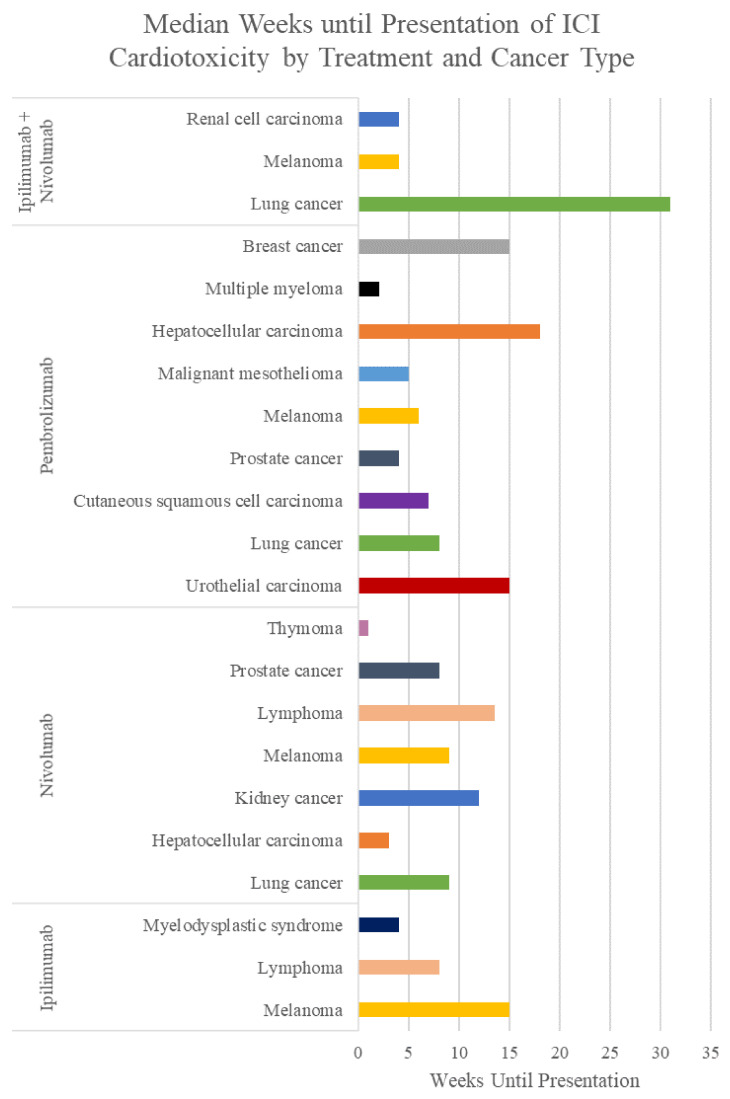
Median weeks until presentation of ICI cardiotoxicity by treatment and cancer type. All cancer types and treatments present within a median of 1–31 weeks after starting treatment.

**Figure 2:**
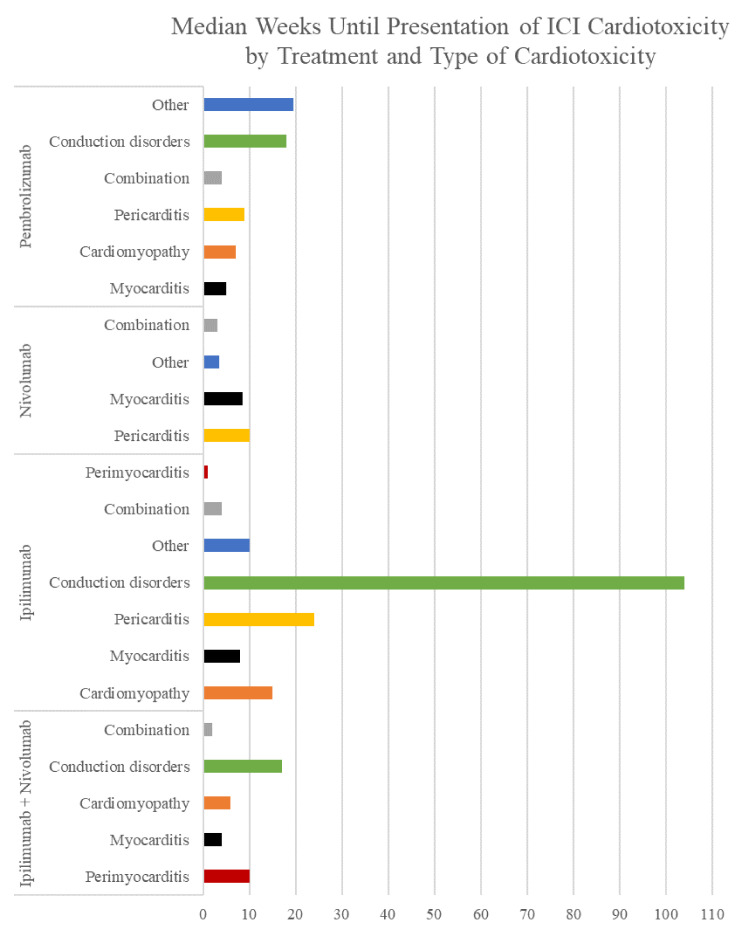
Median weeks until presentation of ICI cardiotoxicity by treatment and type of cardiotoxicity. All types of cardiotoxicity and treatments present within a median of 1–104 weeks after starting treatment.

Patients can have a wide range of signs and symptoms, from asymptomatic to severe chest pain, dyspnea, multiorgan failure, and sudden death [27] (Appendix A). These symptoms usually begin within the first 3 months of starting immunotherapy, but they can also start up to a year after therapy finishes [27]; the average time until symptoms start varies between the treatment (Table 2), the patient’s cancer (Table 3, Figure 1), and the type of cardiotoxicity (Table 4, Figure 2).

The mechanism of cardiotoxicity is still under research, but it may primarily involve CD4+ mediated T cell inflammation [28]. A study by Tay et al. confirmed that nivolumab does not induce cardiomyocyte apoptosis like a cytotoxic drug such as doxorubicin does—rather, it increases pro-inflammatory cytokine production in CD4+ T cells only [29]. The most common cytokines produced include TNF-α, granzyme B, and IFN-γ [27]. Expression of the inflammatory transcription factors NLRP3, MyD88, and p65/NF-κB are also increased in cardiomyocytes after ICI treatment [30]. Further proving this point, anti-PD-1-treated mice had more CD4+ and CD8+ T cell infiltration in the heart as compared to a control group [29]. However, it is unclear exactly what the T cells are targeting.

Reinforcing the idea that T cells are the primary mediators of cardiotoxicity with ICIs, a remarkable study by Wang et al. shows that fatal myocarditis was developed in mice genetically predisposed to systemic autoimmunity due to PD-1 deficiency [31]. However, in mice, the myocarditis was caused by CD4+ and CD8+ T cells as well as autoantibodies against cardiomyocytes. Mice with a genetic predisposition to autoimmunity but without a PD-1 deficiency did not develop myocarditis, demonstrating that prevention of myocarditis is likely mediated by PD-1 [31]. Love et al. found in a similar study that CTLA-4 removal on T cells also caused severe myocarditis in mice, but lack of IL-12 prevented CD8+ T cells from proliferating, thus ameliorating the myocarditis [32]. PD-L1 expressed in the human myocardium is involved in protecting immune-mediated cardiac injury and inflammation [29].

Autoantibodies have been postulated to lead to cardiotoxicity in patients treated with ICIs. A case described by Martinez-Calle et al. showed that the patient did have IgG autoantibodies against cardiac troponin T, but it is unknown if the antibodies were present before initiation of ICI therapy [33]. Two other patients have shown autoantibody deposition in cardiac muscle, suggesting a direct relationship between the antibodies and myocarditis [34]. However, many more cases have stated that no autoantibodies were found on histology or blood tests [11,15,35], leading to the conclusion that autoantibodies are generally not involved in the pathogenesis of cardiotoxicity with ICIs.

### 3.2. Myocarditis

Myocarditis, or inflammation of the myocardial muscle, is the most common form of cardiotoxicity caused by ICIs. It is also the most fatal form of cardiotoxicity, with a mortality rate of up to 50% [16,36,37]. It was first recognized as an IRAE during clinical trials, and as use of ICIs has increased, awareness has also increased.

Myocarditis can develop as early as 2 weeks after starting ICI therapy [17]; however, the median amount of time after beginning ICI treatment that patients experience symptoms is 65 days [18]. Eighty-one percent of patients present within 3 months of initiation of therapy [17]. Patients with ICI-associated myocarditis can present with a wide range of symptoms. Most commonly, patients will have a primary complaint of shortness of breath [15,19]. Palpitations and signs of congestive heart failure, such as edema, fatigue, weakness, or wheezing, are also common symptoms [18].

On lab tests, patients will often experience elevation of BNP or NT-proBNP, signs of active inflammation such as elevated CRP and hepcidin, and elevated troponin and CK-MB [17,20]. Troponin is an important predictor of the patient’s risk of a major adverse cardiac event (MACE), which is defined as cardiovascular death, cardiac arrest, complete heart block, or cardio-genic shock [11,37]. Troponin I is the most useful troponin for this purpose; troponin T may be elevated with concurrent myositis, reducing its usefulness [37]. Patients who do not have an elevated troponin I almost never have myocarditis, making it a very sensitive tool, though it is not specific [38]. NT-proBNP is less specific for myocarditis and can be elevated due to cancer-related inflammation rather than ICI-associated cardiotoxicity [16,39]. An abnormal ECG is also common but is not always present [17].

The gold standard to diagnose myocarditis is endomyocardial biopsy [40,41]. The biopsy needs to be taken from about six regions because the area of inflammation is localized to specific regions in the heart [21,40]. Patients have been reported to have a wide range of histological findings suggestive of myocarditis. As stated earlier, autoantibodies are almost never found in these patients [15]. Histopathological evaluation of endomyocardial biopsies has revealed a lymphocytic infiltrate of CD4+ and CD8+ cells in the myocardium and conducting system of patients with myocarditis [11,19]. There may also be fibrosis without granulomas or histiocytes [17,26]. These T cell populations are clonally similar to those found in tumors as well, suggesting that cardiotoxicity is caused by antigen similarity; however, it may also be caused by reduced inhibition of self-reactive T cells [27]. PD-L1 is also highly expressed in the myocardium of these patients [19]. One of the youngest cases of pembrolizumab-induced myocarditis, described by Läubli et al. [42], similarly demonstrated predominant infiltration of CD8+ cells on myocardial biopsy. These findings seem to indicate that the cardiotoxicity is T-cell-mediated rather than antibody-mediated.

However helpful endomyocardial biopsy may be, this procedure is invasive and not routinely performed. A cardiac magnetic resonance scan (CMR) is also very effective and is the gold standard of imaging for myocarditis [21]. On CMR, patients will have active inflammation with increased capillary permeability, possible myocardial ischemia, and late gadolinium enhancement [17,21]. If CMR is unavailable, PET/CT is a good alternative [22].

Several studies have found that patients who receive combination ICI therapy, most commonly ipilimumab and nivolumab, have an increased risk of developing myocarditis as compared to patients only taking one ICI [6,16,39,43,44]. Zhang et al. also report that there is an increased risk of myocarditis with the female sex and older age [6]; other studies disagree, saying that there is no age association and higher risk with the male sex [16,43]. This discrepancy may be due to the fact that cardiac-adverse events have been more commonly reported in male patients than female patients in the past, since females have often been less represented in clinical trials [43]. There is no association of myocarditis with a specific type of cancer [36]. In addition, there is an increased risk of ICI-associated myocarditis with hypertension; tobacco use; and the use of prescription statins, ACE inhibitors, and angiotensin inhibitors [23]. Up to half of all patients with ICI-associated myocarditis will present with other, concurrent IRAEs [23,44]; in particular, up to 25% of patients with myocarditis may have concomitant myositis, and 10% may have concomitant myasthenia gravis [36].

Interestingly, a study by Awadalla et al. shows that patients receiving ICI therapy may have a lower likelihood of developing myocarditis when they have been vaccinated for influenza [45]. The study’s results show that patients with myocarditis were less likely to have received the flu vaccine; in addition, vaccinated patients who did develop myocarditis had lower serum troponin levels and were less likely to have a MACE [45].

PD-1 expression is constitutive in the myocardium and is important for normal immune function, so inhibition of this pathway can lead to severe issues [46]. In one study, Wang et al. showed that PD-1 deficiency in mice causes them to develop fatal autoimmune myocarditis, almost always within 10 weeks after birth [31]. Myocarditis did not develop in mice with a deficiency in immune-regulating molecules not in the PD-1/PD-L1 pathway [31]. However, Love et al. showed that myocarditis can also develop in mice deficient in CTLA-4 [32]. In humans, patients with myocarditis often have high expression of PD-L1 in cardiac tissue, suggesting that PD-L1 is used by the heart to prevent inflammation [37].

### 3.3. Pericarditis

Pericardial disease, or inflammation of the pericardial sac surrounding the heart, is another common form of cardiotoxicity, with an incidence of 0.3% [24,47]. This includes pericarditis, pericardial effusion, and even clinical tamponade [17]. Though it is not as fatal as myocarditis, it has a mortality rate of 13–21% [6,16,19,48]. On average, patients who develop pericarditis do so within 30 days of starting ICI treatment [16], but several cases have been described where pericarditis was diagnosed several months after starting treatment [35,49,50,51]. Often, patients will present with chest pain, shortness of breath, and hemodynamic instability after ICI therapy [16,24]. Shortness of breath is the most common symptom on presentation [23].

Patients will frequently show ECG changes during pericardial disease [16]. In addition, pericardial effusion or thickening may be seen on CT scan, and CMR will show pericardial inflammation and/or fibrosis [25]. Analysis of pericardial effusion fluid shows lymphocytes and plasma cells without evidence of malignant cells or microorganisms causing the symptoms [17,25,50]. There may also be a hemorrhage or fibrinous exudate present [25]. Pericarditis has rarely been reported to occur in conjunction with myocarditis [44,52]; a normal troponin level can rule out this possibility.

Several studies have shown that there is a higher risk of pericardial disease when ICIs are given for non-small-cell lung cancer (NSCLC) [17,39,47,48,53]. It is hypothesized that this could be due to the use of radiotherapy in lung cancer in addition to ICIs, which exposes more shared antigens [39,50]; however, other studies argue that pericardial diseases are also common complications of certain cancers, thus inflating the significance of this result [48]. In addition, pericarditis is more common in males, but there is no increased risk with age [16,22,53]. Inno et al. found that treatment with anti-PD-1 or anti-PD-L1 therapy is more associated with pericardial disease than anti-CTLA-4 therapy [53]. Patients with pericarditis will frequently display other IRAEs in addition to pericarditis, such as hyper- or hypothyroidism, arthritis, or hepatitis [35,50].

Interestingly, Altan et al. demonstrated that T cells infiltrating the pericardium had lower granzyme B expression present, indicating that perhaps cytotoxic granules are not causing pericardial inflammation and damage [25]; instead, this damage may be due to cytokine production. Antibodies are not involved in pathogenesis of this condition [35].

### 3.4. Takotsubo Cardiomyopathy

Takotsubo cardiomyopathy is a form of stress-induced cardiomyopathy that is an uncommon presentation of ICI-associated cardiotoxicity. Patients often begin having symptoms between 15 weeks and 8 months into treatment [17]. It presents as transient cardiac regional wall motion abnormalities, new ECG changes, and elevated troponin and NT-proBNP [17,26]. Clinicians may also see an apical ballooning pattern on echocardiogram [23].

Takotsubo cardiomyopathy may be caused by a direct effect of ICIs; however, it may also be caused by a sudden release of large amounts of catecholamines [44]. In addition, it may be a result of delayed cardiotoxicity from previous rounds of chemotherapy in patients who have not received ICIs as first line therapy [44]. As this is a relatively uncommon cardiotoxicity, there has not been much research on this presentation to date, and the cause is undetermined in the majority of cases.

### 3.5. Conduction Diseases

Conduction diseases caused by ICIs can include atrial fibrillation, ventricular tachycardia or fibrillation, and atrioventricular conduction disorders [17]. These diseases may come in conjunction with myocarditis or may appear separately. All conduction diseases are associated with increased mortality [17,18]; they can frequently cause sudden death as well [39]. The most common conduction disorder is atrial fibrillation [22].

It is unknown what causes conduction disease, but local inflammation or fibrosis may play a role. Other hypotheses include an electrolyte imbalance or non-inflammatory left ventricular dysfunction [22]. Lyon et al. argues that systemic inflammation caused by cancer and ICI treatment may also worsen cardiovascular conditions, leading to arrhythmias [44].

### 3.6. Myocardial Infarction

This extremely rare cardiac complication has been seen in atezolizumab and pembrolizumab treatment. The exact cause of this cardiotoxicity manifestation is unknown, but Chen et al. hypothesize that it may be due to rupture of atherosclerotic plaques seen in chronic inflammatory conditions, coronary spasm, or direct activation of T cells, leading to coronary vasculitis [17]. In addition, a myocardial infarction may be a result of previous cardiac arrhythmias caused by the ICI treatment combined with the hypercoagulability that is seen in patients with advanced cancer.

## 4. Treatments

### 4.1. Ipilimumab

Ipilimumab is a fully human recombinant antibody against CTLA-4 [54]. It was initially approved by the FDA in 2011 and is now used for treatment of multiple cancers, including melanoma, renal cell carcinoma, colorectal cancer, hepatocellular carcinoma, and non-small-cell lung cancer [55] (Table 5). There are no contraindications [55].

Because ipilimumab works by stimulating the immune system, patients do not experience common cytotoxic chemotherapy side effects such as bone marrow suppression; instead, immune-related adverse events develop in up to 90% of patients [8]. The most common adverse effects patients experience while taking ipilimumab include fatigue, diarrhea, pruritis, rash, and colitis; severe immune-related reactions include enterocolitis, hepatitis, dermatitis, neuropathy, and endocrinopathy; it is seen in less than 1% of patients [55]. Ipilimumab very rarely causes cardiotoxicity. These IRAEs are dose-dependent and often happen within the first 3 months of the start of treatment [8]. If a patient experiences a severe IRAE, most physicians recommend permanent discontinuation of the drug. For more minor side effects, the drug may be withheld until the patient improves, but afterward, the patient can continue treatment [55,56].

### 4.2. Pembrolizumab

Pembrolizumab is a humanized IgG4 antibody against PD-1 [57]. This antibody does not trigger antibody-dependent cellular cytotoxicity, unlike normal IgG antibodies [10]. It was initially approved by the FDA in 2014 and is currently approved for treatment of many cancers, including melanoma, lung cancer, several types of squamous cell cancer, several types of lymphomas, urothelial carcinoma, any cancer that is high in microsatellite instability or is mismatch-repair-deficient, gastric and esophageal cancer, cervical cancer, hepatocellular carcinoma, Merkel cell carcinoma, renal cell carcinoma, endometrial carcinoma, tumor mutational burden high cancer, and triple-negative breast cancer (Table 5) [58].

Similar to ipilimumab, pembrolizumab commonly causes immune-related adverse events rather than cytotoxic effects, seen in up to 70% of patients [8]. These events can be severe or even fatal and happen in any body system or organ [58]. The most common adverse events experienced by patients are fatigue, musculoskeletal pain, decreased appetite, diarrhea, rash, fever, cough, constipation, nausea, abdominal pain, and pruritis [58]. Rarely, pembrolizumab can cause cardiotoxicity such as myocarditis and pericarditis, but this occurs in less than 1% of patients [58].

### 4.3. Nivolumab

Nivolumab is a fully human IgG4 antibody against PD-1 [59]. Similar to pembrolizumab, it does not work in the pathway of antibody-dependent cellular cytotoxicity [10]. It was initially approved by the FDA in 2014 and is now approved for treatment of many cancers, including melanoma, non-small-cell lung cancer, malignant pleural mesothelioma, renal cell carcinoma, classical Hodgkin lymphoma, squamous cell carcinoma of the head and neck, urothelial carcinoma, colorectal cancer, hepatocellular carcinoma, and esophageal squamous cell carcinoma (Table 5) [60].

In patients, nivolumab can cause a number of side effects related to immune cell overactivation, which can be severe. The most common adverse effects seen are fatigue, rash, pruritis, and diarrhea [1]; other common symptoms include musculoskeletal pain, nausea, vomiting, abdominal pain, constipation, cough, dyspnea, asthenia, upper respiratory tract infections, fever, and headache [60]. These IRAEs can develop very late after finishing ICI treatment because a single administration of nivolumab can cause inactivation of PD-1 molecules for almost 3 months [61]. Cardiac adverse effects have been seen in less than 1% of patients [60].

Ipilimumab is often administered with nivolumab for treatment of advanced renal cell carcinoma, microsatellite instability-high or mismatch repair deficient metastatic colon cancer, hepatocellular carcinoma, and metastatic non-small-cell lung cancer (even if PD-L1 expression is less than 1%) [55,62]. However, side effects of this combination can be severe, leading to discontinuation of therapy in up to 40% of patients [46]. Though cardiotoxicity is still rare, combination ipilimumab and nivolumab therapy has a higher incidence of cardiac-adverse effects than either treatment alone [11].

### 4.4. Atezolizumab

Atezolizumab is a humanized IgG mouse antibody against PD-L1 [63]. It was initially approved by the FDA in 2016 and has since been approved for urothelial carcinoma, non-small-cell lung cancer, triple-negative breast cancer, small-cell lung cancer, hepatocellular carcinoma, and melanoma (Table 5) [64]. The most common reported adverse effects include fatigue, nausea and vomiting, cough, dyspnea, decreased appetite, alopecia, constipation or diarrhea, headache, and rash; the specific side effects vary based on the cancer being treated [64]. Myocarditis or pericarditis are less common side effects occurring in less than 1% of patients; for any grade severity of cardiotoxicity, the drug is recommended to be permanently discontinued [63,64]. To the best of our knowledge, only one case has been reported in the literature about cardiotoxicity using atezolizumab specifically [65]. More case reports may be seen in the future as this drug continues to be used more frequently as a first-line biologic agent.

### 4.5. Durvalumab

Durvalumab is a fully human IgG antibody against PD-L1 that was initially approved by the FDA in 2017 [66,67]. It is indicated for the treatment of urothelial carcinoma after platinum-containing therapy and non-small-cell lung cancer (Table 5) [66]. The most common side effects reported include fatigue, constipation, urinary tract infections, edema, pneumonitis, dyspnea, rash, cough, and nausea, depending on the patient’s cancer type [66]. More severe side effects include hyperthyroidism or hypothyroidism, colitis, diarrhea, and hepatitis, which can be fatal [67]. With high-grade side effects, the drug may need to be permanently discontinued, but usually the drug only needs to be halted or the dose lowered [67]. Cardiotoxicity for this drug is very rare and has only been reported in three cases by name [19,20,38]. Expanded use of this drug may reveal more significant cardiotoxicity in the future.

## 5. Antidotes for ICI-Associated Cardiotoxicity

The most frequently recommended treatment for ICI-associated cardiotoxicity is high-dose corticosteroids [68,69]. Especially for myocarditis, higher doses of corticosteroids have been associated with better outcomes for patients [20]. Patel et al. recommend that patients receive 1000 mg/day of corticosteroids initiated within 24 h of presentation; after resolution of symptoms, steroids should be tapered over at least 4–6 weeks [23].

However, there have also been cases of cardiotoxicity that are refractory to steroids. In these cases, Brahmer et al. recommend cardiac transplant rejection medications, including mycophenolate mofetil, infliximab, or anti-thymocyte globulin [68]. However, infliximab cannot be given to patients with heart failure [6]. In addition, other symptom-based treatments are often used, such as anti-coagulants, beta blockers or ACE inhibitors in heart failure, amiodarone with arrhythmias, and pericardiocentesis or pericardial window placement in cardiac tamponade [22]. For pericarditis specifically, Waliany et al. recommend that NSAIDs and colchicine are also effective in treating signs and symptoms [7].

Several studies have recommended that even after minor grade cardiotoxicity, ICIs need to be discontinued temporarily; in high-grade toxicities, they must be permanently discontinued [56]. However, ICIs have a long half-life in the body; therefore, stopping treatment will not immediately reverse the biological effect of the drug [44]. Due to this pharmacological property, steroid treatment often has to be tapered over several weeks to ensure that the patient’s condition does not worsen after halting treatment.

To prevent severe cardiotoxicity in the future, several studies recommend routine cardiac surveillance before starting ICI treatment and within the first 1–4 cycles or up to 12 weeks into treatment [39,44,70]. Lyon et al. recommends testing NT-proBNP, cardiac troponin, and an ECG for this purpose [44]. This surveillance is particularly important in patients with pre-existing cardiac problems [71,72]. In order to carry out this surveillance, baseline cardiac function testing needs to be performed, because patients with preexisting cardiac problems may have high troponin levels due to that disease process rather than ICI cardiotoxicity [73]. Sarocchi et al. report that, during a trial of monitoring troponin levels to predict myocarditis, a few patients developed a small elevation of troponin levels without any symptoms related to this increase; because of this, they postulate that many patients may have subclinical myocarditis [73].

## 6. Discussion

Cardiotoxicity, though a rare side effect of immune checkpoint inhibitors, is a concerning one due to its high mortality rate and presentation soon after treatment initiation. The most common cardiotoxicity, myocarditis, has by far the highest mortality rate of all cardiotoxicities. However, if they do not progress to become fatal, these cardiotoxicities often resolve quickly with steroid treatment.

There is much variability in the weeks until cardiotoxicity presentation between the type of cardiotoxicity, the specific antibody, and the type of cancer being treated. As shown by the averages and medians in Table 2, Table 3 and Table 4, the bell curve of weeks until presentation of cardiotoxicity is positively skewed. This indicates that though the majority of cases present soon after initiation of ICI treatment, there are several cases that have presented as far as 2 years after initiation of treatment. Based on this information, clinicians must be fully vigilant regarding cardiac toxicity in patients soon after treatment initiation. However, the risk of cardiotoxicity never completely disappears; patients may need to be monitored for suspicious symptoms even years after starting ICI treatment.

Between treatments, ipilimumab appears to be the treatment that presents with cardiotoxicity the latest, and ipilimumab + nivolumab patients present in the shortest time (excluding atezolizumab due to lack of corroborating data). It is unclear why this is the case. However, several studies have stated that the PD-1/PD-L1 pathway specifically is important in preventing cardiac autoimmunity [4,5,46,74], so the CTLA-4 pathway may be less important and thus take longer to cause cardiotoxicity. In addition, a combination of ICIs may cause a faster presentation of immune-related adverse events in general due to more widespread blockage of inhibitory signals.

Within cancer types, there is much variability as well, but there does not appear to be a clear pattern in terms of which cancer presents with cardiotoxicity in a shorter or longer timeframe. Within types of cardiotoxicity, however, clearer trends are seen. Patients who present with a combination of cardiotoxicities frequently present very early into treatment as compared to single cardiotoxicities. In addition, myocarditis tends to present in a shorter time frame than many other cardiotoxicity types. It is unclear why this trend appears. Additional research needs to be done to confirm these trends and investigate why this occurs.

Further research also needs to be done on myocardial infarctions and Takotsubo cardiomyopathy in patients taking immune checkpoint inhibitors. These presentations are uncommon but can be fatal, and the timing, histopathological findings, and symptoms of patients with these conditions due to ICI treatment is poorly understood. In addition, very few case reports were found for patients with cardiotoxicity taking durvalumab or atezolizumab; as these drugs become more widely used, investigations into the likelihood of cardiotoxicity with these antibodies need to be carried out.

## 7. Conclusions

Immune checkpoint inhibitors may cause several immune-related side effects; one rare side effect is cardiac toxicity, including myocarditis, pericarditis, Takotsubo cardiomyopathy, arrhythmias, and/or myocardial infarction. Though these side effects are rare, they have a high mortality rate; it is important to be aware of the common symptoms and lab results associated with them while treating patients. The best way to treat any cardiac toxicity is usually high doses of steroids; other symptomatic treatments may also be warranted.

## Figures and Tables

**Table 1:** ICI-associated cardiotoxicities and their characteristics.

Type of Cardiotoxicity	Median Onset	Most Common Symptoms	Diagnosis	Treatment	Sources
Myocarditis	65 days	Shortness of breath, palpitations, edema, and fatigue	NT-proBNP, troponin I, endomyocardial biopsy, and CMR	High-dose corticosteroids	[15,16,17,18,19,20,21]
Pericarditis	30 days	Shortness of breath, chest pain	ECG changes, CMR, pericardial effusion, and fluid analysis	Pericardiocentesis, NSAIDs, colchicine, and high-dose corticosteroids	[7,16,22,23,24,25]
Takotsubo cardiomyopathy	15 weeks to 8 months	Unknown	ECG changes, troponin and NT-proBNP	High-dose corticosteroids	[17,23,26]
Arrhythmias and conduction disorders	Unknown	Unknown	ECG changes	High-dose corticosteroids, supportive	[17,18,22]
Myocardial infarction	Unknown	Chest pain and shortness of breath	ECG changes, troponin, and others	Cardiac catheterization, supportive	[17]

Abbreviations: ICI, immune checkpoint inhibitor; NT–proBNP, N-terminal -prohormone brain natriuretic peptide; CMR, cardiovascular magnetic resonance imaging; ECG, electrocardiogram; NSAIDs, non-steroidal anti-inflammatory drugs.

**Table 2:** Weeks until Presentation of ICI-associated cardiotoxicity, organized by treatment.

Treatment	Ipilimumab	Nivolumab	Pembrolizumab	Ipilimumab + Nivolumab	Durvalumab	Atezolizumab	Unspecified
Number of Cases	18	43	20	17	3	1	5
Average Weeks until Presentation	18	17	9	10	11	1	--
Median Weeks until Presentation	10	9	7	6	8	1	--

**Table 3:** Weeks until ICI-associated cardiotoxicity, organized by treatment and type of cancer.

Treatment	Type of Cancer Treated	Number of Cases	Average Weeks until Presentation	Median Weeks until Presentation
Ipilimumab	Melanoma	13	21	15
	Lymphoma	1	8	8
	MDS	2	4	4
Nivolumab	Lung Cancer	30	20	9
	HCC	1	3	3
	Kidney Cancer	1	12	12
	Melanoma	7	12	9
	Lymphoma	2	14	14
	Prostate Cancer	1	8	8
	Thymoma	1	1	1
Pembrolizumab	Urothelial Carcinoma	1	15	15
	Lung Cancer	7	7	8
	Cutaneous SCC	1	7	7
	Prostate Cancer	1	4	4
	Melanoma	6	9	6
	Malignant Mesothelioma	1	5	5
	HCC	1	18	18
	Multiple Myeloma	1	2	2
	Breast Cancer	1	15	15
Ipilimumab + Nivolumab	Lung Cancer	2	31	31
	Melanoma	13	8	4
	RCC	2	4	4

Abbreviations: MDS, myelodysplastic syndrome; HCC, hepatocellular carcinoma; SCC, squamous cell carcinoma; and RCC, renal cell carcinoma.

**Table 4:** Weeks until ICI-associated cardiotoxicity, organized by treatment and type of cardiotoxicity.

Treatment	Type of Cardiotoxicity	Number of Cases	Average Weeks until Presentation	Median Weeks until Presentation
Ipilimumab + Nivolumab	Perimyocarditis	1	10	10
	Myocarditis	11	10	4
	Cardiomyopathy	3	13	6
	Conduction Disorders	1	17	17
	Combination	1	2	2
Ipilimumab	Perimyocarditis	1	1	1
	Myocarditis	7	12	8
	Cardiomyopathy	3	15	15
	Conduction Disorders	1	104	104
	Pericarditis	3	23	24
	Combination	1	4	4
	Other	1	10	10
Nivolumab	Myocarditis	10	11	9
	Pericarditis	21	22	10
	Combination	6	4	3
	Other	6	10	4
Pembrolizumab	Myocarditis	7	6	5
	Cardiomyopathy	1	7	7
	Conduction Disorders	1	18	18
	Pericarditis	5	10	9
	Combination	4	5	4
	Other	2	20	20

**Table 5:** Immune checkpoint inhibitors, their indications, and side effects.

Drug	First Approved	Cancers Approved for Treatment	Most Common Side Effects
Ipilimumab	2011	Melanoma, RCC, CRC, HCC, and NSCLC	Fatigue, diarrhea, pruritis, rash, and colitis
Pembrolizumab	2014	Melanoma, lung cancer, SCC, lymphomas, urothelial carcinoma, cancers high in MSI, MMR-deficient cancers, gastric cancers, esophageal cancers, cervical cancers, HCC, Merkel cell cancer, RCC, endometrial carcinoma, tumor mutational burden-high cancer, and triple-negative breast cancer	Fatigue, musculoskeletal pain, decreased appetite, diarrhea, rash, fever, cough, constipation, nausea, abdominal pain, and pruritis
Nivolumab	2014	Melanoma, NSCLC, malignant pleural mesothelioma, RCC, classic Hodgkin lymphoma, HNSCC, urothelial carcinoma, CRC, HCC, and esophageal squamous cell carcinoma	Fatigue, rash, pruritis, and diarrhea
Atezolizumab	2016	Urothelial carcinoma, NSCLC, triple-negative breast cancer, SCLC, HCC, and melanoma	Fatigue, nausea, vomiting, cough, dyspnea, decreased appetite, alopecia, constipation or diarrhea, headache, and rash
Durvalumab	2017	Urothelial carcinoma and NSCLC	Fatigue, constipation, UTIs, edema, pneumonitis, dyspnea, rash, cough, and nausea

Abbreviations: RCC, renal cell carcinoma; CRC, colorectal cancer; HCC, hepatocellular carcinoma, NSCLC, non-small-cell lung cancer; SCC, squamous cell carcinoma; MSI, microsatellite instability; MMR, mismatch repair; HNSCC, head and neck squamous cell carcinoma; SCLC, small-cell lung cancer; and UTIs, urinary tract infections.

## Abbreviations

ICI	Immune checkpoint inhibitor
IRAE	Immune-related adverse event
MACE	Major adverse cardiac event
CMR	Cardiovascular magnetic resonance imaging
NSCLC	Non-small-cell lung cancer

## Appendix A

**Table A1:** Patients with a wide range of signs and symptoms, from asymptomatic to severe chest pain, dyspnea, multiorgan failure, and sudden death.

Study	Sex	Age	Type of Cancer	Cancer Treatment	Type of Cardiotoxicity	Weeks until Presentation	Complaint at Presentation	Antidote
[38]	M	76	Lung cancer	Durvalumab	Myocarditis	8 weeks	NR	Prednisone
[38]	F	67	Renal cell carcinoma	Ipilimumab + nivolumab	Myocarditis	2 weeks	Dyspnea	Dexamethasone
[38]	M	82	Urothelial carcinoma	Pembrolizumab	Myocarditis	15 weeks	Chest pain and dyspnea	Methylprednisone and prednisone
[19]	M	70	Lung cancer	Nivolumab	Acute pericarditis and pericardial effusion	13 weeks	Pleuritic chest pain	Prednisone
[19]	F	60	Lung cancer	Nivolumab and THU-decitabine	Acute pericarditis and pericardial effusion	9 weeks	Pleuritic chest pain, shortness of breath, productive cough	Colchicine and ibuprofen
[19]	M	58	Non-small cell lung cancer	Nivolumab	Pericardial effusion	10 weeks	Shortness of breath	Prednisone
[19]	M	60	Melanoma	Ipilimumab + nivolumab, then nivolumab	Cardiomyopathy	22 weeks	Chest tightness, decreased exercise tolerance	Prednisone
[19]	F	84	Lung cancer	Pembrolizumab	Cardiomyopathy	7 weeks	Shortness of breath	Heart failure treatment
[19]	F	71	Lung cancer	Durvalumab	Cardiomyopathy and pericardial effusion	22 weeks	Abdominal pain, shortness of breath on exertion	Prednisone
[19]	M	67	Melanoma	Ipilimumab	Cardiomyopathy	17 weeks	Intermittent chest discomfort, shortness of breath on exertion	Carvedilol
[19]	F	80	Cutaneous squamous cell carcinoma	Pembrolizumab	Myocarditis	7 weeks	Bilateral eye ptosis, generalized weakness and fatigue	Prednisone
[19]	M	80	Prostate cancer	Pembrolizumab	Myocarditis	4 weeks	Right eye ptosis, generalized weakness and fatigue	Prednisone
[75]	M	75	Myelodysplastic syndrome	Ipilimumab + nivolumab + azacitidine	Pericardial effusion and pleural effusion	7 weeks	Fever, cough, dyspnea, rash	IVIG, rosuvastatin
[75]	M	78	Myelodysplastic syndrome	Ipilimumab and azacitidine	Perimyocarditis	1 week	Fever and pneumonia	IVIG, colchicine, atorvastatin
[75]	F	74	Melanoma	Ipilimumab and evofosfamide	Paroxysmal atrial fibrillation, sinus tachycardia, myocarditis	4 weeks	Dyspnea, fever, hypotension, rash	IVIG, colchicine, rosuvastatin, hydroxychloroquine
[52]	M	62	Hepatocellular carcinoma	Nivolumab	Perimyocarditis, takotsubo syndrome, myocardial infarction	3 weeks	Chest pain, nausea, and vomiting	Methylprednisolone, prednisone, broad spectrum antibiotics
[49]	M	58	Non-small cell lung cancer	Nivolumab	Pericarditis	78 weeks	limb edema	Methylprednisolone, prednisolone, infliximab
[76]	F	58	Lung cancer	Nivolumab	Pericarditis	12 weeks	dyspnea and cough	Pericardiocentesis and corticosteroids
[76]	M	65	Lung cancer	Nivolumab	Pericarditis	106 weeks	Acute respiratory failure	Corticosteroids and pericardiocentesis
[74]	F	78	Melanoma	Pembrolizumab	Myocarditis	6 weeks	Chest pain	Prednisone
[74]	F	55	Melanoma	Nivolumab, then ipilimumab + nivolumab	Myocarditis	4 weeks after ipilimumab + nivolumab	Chest pain, fever, and dyspnea	
[77]	F	52	Non-small cell lung cancer	Ipilimumab + nivolumab	Myocarditis and acute heart failure	52 weeks	Dyspnea on exertion, chest pain, and lower extremity edema	Methylprednisolone
[78]	M	62	Lung cancer	Pembrolizumab	Pericarditis	15 weeks	Shortness of breath	Pericardial window and prednisone
[79]	M	62	Lung cancer	Nivolumab	Cardiac tamponade	1 week	Dyspnea	Pericardiocentesis
[80]	F	66	Lung cancer	Pembrolizumab	Pericardial effusion	1 week	NR	Pericardiocentesis and prednisone
[81]	M	68	Melanoma	Ipilimumab + nivolumab	Myocarditis	6 weeks	Dyspnea, irregular heartbeats, tachycardia	Prednisone and solumedrol
[82]	F	66	Lung cancer	Nivolumab	Myocarditis	9 weeks	Chest pain	Methylprednisolone, plasmapheresis, abatacept
[83]	F	71	Melanoma	Pembrolizumab	Myocarditis and cardiac arrhythmia	6 weeks	Dyspnea	Methylprednisolone, mycophenolate mofetil, plasmapheresis, rituximab, alemtuzumab
[84]	F	79	Lung cancer	Pembrolizumab	Pericarditis	9 weeks	Chest pain	Pyridostigmine and methylprednisolone
[25]	M	72	Lung cancer	anti-PD-L1	Pericarditis	11 weeks	Dyspnea, hypotension, hypoxia	NR
[25]	F	65	Lung cancer	anti-CTLA-4 and anti-PD-1	Arrhythmias	19 weeks	Loss of consciousness and hypotension	Pacemaker
[25]	M	57	Lung cancer	anti-PD-L1	Cardiac tamponade	14 weeks	Dyspnea, orthopnea, bilateral lower extremity edema	NR
[85]	M	80	Kidney cancer	Nivolumab	Myocarditis and atrial fibrillation	12 weeks	Severe asthenia	Methylprednisolone
[86]	F	78	Melanoma	Nivolumab	Myocarditis	4 weeks	Muscle weakness and dyspnea	Methylprednisolone to pulse steroid
[65]	F	61	Lung cancer	Atezolizumab	Myocarditis	Less than 1 week	Dyspnea, fatigue	Methylprednisolone and mycophenolate mofetil
[87]	F	55	Melanoma	Nivolumab	Myocarditis	6 weeks	Dysphagia, dyspnea, limb weakness	IGI, steroid pulse, plasma exchange
[88]	M	74	Lung cancer	Nivolumab	Myocardial necrosis	6 weeks	General malaise, appetite decrease, dyspnea	Catecholamines
[89]	F	76	T cell lymphoma	Brentuximab and nivolumab	Acute heart failure	3 weeks	Fatigue, dyspnea, orthopnea	Solumedrol, Impella implant
[90]	M	33	Hodgkin lymphoma	Nivolumab	Complete heart block, myocarditis	24 weeks	NR	Mycophenolate mofetil and steroids
[91]	M	73	Malignant mesothelioma	Pembrolizumab	Myocarditis	5 weeks	Progressive dyspnea and fatigue	Prednisolone, pacemaker, IGI, and plasmapheresis
[91]	M	89	Melanoma	Pembrolizumab	Myocarditis	3 weeks	Weakness, myalgias, and dyspnea	Methylprednisolone, oral prednisone, anti-thymocyte globulin
[91]	F	65	Lung cancer	Nivolumab	Acute coronary syndrome, acute decompensated heart failure	1 week	Dyspnea, edema, bradycardia	Methylprednisolone, prednisone, furosemide, anti-thymocyte globulin
[91]	M	67	Melanoma	Nivolumab	Myocarditis	9 weeks	Chest pain and palpitations	prednisone, infliximab, oral corticosteroids
[92]	M	42	Hepatocellular carcinoma	Pembrolizumab	Bradycardia	18 weeks	Fatigue, dizziness, and anorexia	Cortisone
[93]	F	47	Melanoma	Ipilimumab + nivolumab, then nivolumab	Heart failure, asymptomatic supraventricular tachycardia	17 weeks	Dyspnea, tachycardia, and pulmonary edema	Methylprednisolone and infliximab
[76]	F	58	Lung cancer	Nivolumab	Pericardial effusion	12 weeks	Dyspnea and cough	Pericardiocentesis and steroids
[76]	M	65	Lung cancer	Nivolumab	Pericardial effusion	106 weeks	Acute respiratory failure and fever	Surgical drainage and steroids
[94]	M	70	Lung cancer	Pembrolizumab	Cardiac tamponade	9 weeks	Dyspnea and general fatigue	Pericardiocentesis
[95]	M	65	Lung cancer	Nivolumab	Cardiac tamponade	8 weeks	Dyspnea	Pericardiocentesis
[95]	M	71	Lung cancer	Nivolumab	Cardiac tamponade	6 weeks	Chest pain and dyspnea	Pericardiocentesis
[76]	F	55	Lung cancer	Nivolumab	Pericardial effusion	9 weeks	None	None
[96]	M	71	Lung cancer	Nivolumab	Pericardial effusion	5 weeks	NR	Pericardiocentesis and pericardial window
[97]	M	79	Prostate cancer	Nivolumab	Myocarditis	8 weeks	Blurred vision, pain and stiffness in the upper back	Methylprednisolone, oral prednisone taper
[98]	F	70	Lung cancer	Nivolumab	Pericardial effusion	Less than 1 week	Chest pain and shortness of breath	Colchicine and prednisone
[99]	M	74	Lung cancer	Pembrolizumab	Myocarditis and arrhythmia	8 weeks	Dyspnea on exertion	Prednisone, aspirin, clopidogrel, IV heparin, metoprolol succinate
[69]	M	77	Melanoma	Ipilimumab	Myocarditis	NR	Malaise, nausea, cough, bradycardia	Methylprednisolone
[33]	F	67	Multiple myeloma	Pembrolizumab	Myocarditis	2 weeks	Dyspnea and malaise	Methylprednisolone
[100]	M	52	Renal cell carcinoma	Ipilimumab + nivolumab	Myocarditis	6 weeks	None	Beta blocker therapy
[101]	M	67	Melanoma	Ipilimumab + nivolumab	Acute decompensated heart failure, arrhythmia, chronic heart failure	2 weeks	Dyspnea and cough	Methylprednisolone, anti-thymocyte globulin, and permanent pacemaker implantation
[50]	M	69	Lung cancer	Nivolumab	Pericarditis, pericardial tamponade	73 weeks	Dyspnea, tachycardia, and fever	Prednisone
[102]	F	45	Melanoma	Ipilimumab + nivolumab	Acute heart failure, Takotsubo-like syndrome	Less than 1 week	NR	Methylprednisolone
[102]	M	77	Melanoma	Ipilimumab, nivolumab	Takotsubo-like syndrome	6 weeks	NR	Methylprednisolone
[21]	F	41	Melanoma	Ipilimumab + nivolumab	Myocarditis	9 weeks	Dyspnea	Methylprednisolone
[103]	M	60	Melanoma	Nivolumab	Myocarditis	39 weeks	Fatigue and fever	Prednisolone, IGI
[20]	F	75	Extraskeletal myxoid carcinoma	Durvalumab and tremelimumab	Myocarditis, heart failure, complete heart block	3 weeks	Difficulty ambulating and dyspnea	Methylprednisolone, mycophenolate mofetil
[104]	F	55	Breast cancer	Pembrolizumab	Pericardial tamponade	15 weeks	Pericardial chest pain	Anterior pericardectomy, corticosteroids
[105]	F	76	Lung cancer	Nivolumab	Myocarditis, complete atrioventricular block	3 weeks	Dyspnea	Methylprednisolone and infliximab
[106]	M	72	Melanoma	Ipilimumab + nivolumab	Myocarditis	20 weeks	Dyspnea, leg edema	Prednisolone
[107]	M	73	Lung cancer	Pembrolizumab	Complete atrioventricular block and myocarditis	2 weeks	Faintness	Methylprednisolone and temporary pacemaker implantation
[108]	M	43	Thymoma	Nivolumab	Myocarditis	1 week	Chest discomfort, fatigue, lower limb myalgias	IGI, methylprednisolone
[109]	M	55	Lung cancer	Nivolumab	Acute decompensated right-sided heart failure and cardiogenic shock	1 week	Lethargy and dyspnea	NR
[110]	F	49	Melanoma	Ipilimumab + nivolumab	Myocarditis	2 weeks	Atypical chest discomfort at the cardiac apex	Methylprednisolone and IGI
[111]	F	35	Melanoma	Ipilimumab	Myocarditis	2 weeks	Progressive dyspnea	Methylprednisolone, IGI, plasma exchanges
[112]	M	61	Lung cancer	Nivolumab	Acute coronary syndrome	33 weeks	NR	Corticosteroids
[113]	M	60	Lung cancer	Nivolumab	Pericarditis	17 weeks	NR	Pericardiocentesis and methylprednisolone
[114]	M	68	Histiocytosis and left buttock sarcoma	Anti-PD-L1 and anti-CTLA-4 (unspecified)	Myocarditis and arrhythmia	2 weeks	Fatigue, general malaise, weakness	Steroids, mycophenolate mofetil, temporary transvenous pacing wire followed by a permanent pacemaker
[115]	M	54	Lung cancer	Nivolumab	Heart failure	4 weeks	Dizziness, nausea, loss of consciousness, general paralysis	High dose steroids and a pacemaker
[116]	M	63	Melanoma	Nivolumab	Atrioventricular block, myocardial infarction	3 weeks	Dyspnea, dysphagia, worsened muscle pain	Prednisone and antibiotic therapy (sultamicillin), aspirin, and unfractionated heparin
[117]	M	59	Lung cancer	Nivolumab	Cardiac tamponade	9 weeks	NR	Pericardiocentesis, prednisone, and anti-tubercular treatment
[118]	M	67	Lung cancer	Nivolumab	Cardiac tamponade	15 weeks	Acute respiratory failure	Pericardiocentesis and prednisone
[35]	F	65	Melanoma	Ipilimumab	Cardiac tamponade	38 weeks	Shortness of breath and chest discomfort	Pericardiocentesis and methylprednisolone
[119]	M	46	Small cell lung cancer	Nivolumab	Cardiac tamponade	9 weeks	NR	Pericardiocentesis
[119]	F	54	Lung cancer	Nivolumab	Cardiac tamponade	7 weeks	NR	Pericardiocentesis and prednisone
[11]	F	65	Melanoma	Ipilimumab + nivolumab	Myocarditis	2 weeks	Atypical chest pain, dyspnea, fatigue	Methylprednisolone
[11]	M	63	Melanoma	Ipilimumab + nivolumab	Myocarditis	2 weeks	Fatigue and myalgias	Methylprednisolone and infliximab
[120]	M	60	Melanoma	Ipilimumab	Atrial fibrillation	104 weeks	None	Lisinopril, metoprolol changed to carvedilol
[121]	F	68	Melanoma	Ipilimumab and nivolumab	Myocarditis	2 weeks	Right eye ptosis, generalized weakness and fatigue	Steroids
[122]	M	73	Melanoma	Ipilimumab and nivolumab followed by pembrolizumab	Myocarditis with cardiomyopathy and ventricular arrhythmia	2 weeks after starting pembrolizumab	NR	Prednisolone
[122]	M	87	Melanoma	Nivolumab	Asystole	17 weeks	Cardiac arrest	Prednisolone
[122]	M	77	Melanoma	Ipilimumab followed by penbrolizumab	Stable angina pectoris	10 weeks	Stable angina pectoris	None
[123]	F	69	Melanoma	Nivolumab	Myocarditis	9 weeks	General malaise and palpitations	Prednisolone
[26]	M	72	Melanoma	Ipilimumab	Myocarditis	9 weeks	Dyspnea and anasarca	Corticosteroids
[26]	M	68	Melanoma	Ipilimumab	Cardiomyopathy	12 weeks	Dyspnea and lower extremity edema	Diuresis and coronary catheterization
[26]	M	71	Melanoma	Ipilimumab	Myocardial fibrosis	6 weeks	No obvious cardiac symptoms	High dose steroids
[26]	M	81	Melanoma	Ipilimumab	Heart failure, myocarditis	20 weeks	Progressive subacute dyspnea	Diuretics
[26]	M	23	Melanoma	Ipilimumab	Myocarditis and heart failure	30 weeks	Chest pain and cough	Methylprednisolone converted to prednisone
[26]	M	64	Melanoma	Ipilimumab	Myocarditis	6 weeks	Fatigue, seizures, and abdominal pain (Yun et al. 2015)	Dopamine and fentanyl
[26]	M	88	Melanoma	Pembrolizumab	Cardiac arrest	24 weeks	Myalgia and pain in the shoulder	Corticosteroids
[26]	M	80	Non-Hodgkin lymphoma	Ipilimumab	Myocarditis	8 weeks	Dyspnea, edema, and arrhythmia	Methylprednisolone and prednisone
[124]	M	64	Lung cancer	Nivolumab	Cardiac tamponade	13 weeks	Pericardial chest pain	Pericardiocentesis and pericardial window
[125]	M	75	Lung cancer	Nivolumab	Myocarditis	18 weeks	Acute dyspnea and chest pain	Prednisolone, ACE inhibitor, beta blocker, diuretic therapy
[126]	F	68	Lung cancer	Nivolumab	Myocarditis and arrhythmia	3 weeks	Altered mental status, nausea, and vomiting	Methylprednisolone and amiodarone IV
[127]	F	83	Melanoma	Ipilimumab	Takotsubo cardiomyopathy	15 weeks	Substernal chest pain and dyspnea	Beta blocker therapy
[42]	F	73	Melanoma	Pembrolizumab	Acute heart failure	15 weeks	Progressive dyspnea	AT-2 receptor blocker, beta blocker, spironolactone, and diuretics
[128]	M	59	Melanoma	Ipilimumab	Acute fibrinous pericarditis	24 weeks	Chest pain and dyspnea	Methylprednisolone, prednisone, budesonide
